# Relationship Between Postoperative Pain in Surgery and the Onset of Psychological Symptoms Such as Mental Anxiety and Depression

**DOI:** 10.62641/aep.v54i3.2216

**Published:** 2026-06-15

**Authors:** Yunyun Chen, Wenting Hu, Liangliang Tan, Jing Ren, Xiang Bao

**Affiliations:** ^1^Department of Anorectal, Zhongda Hospital, Southeast University, 210009 Nanjing, Jiangsu, China; ^2^Urology Department, Zhongda Hospital, Southeast University, 210009 Nanjing, Jiangsu, China; ^3^Department of Psychosomatics and Psychiatry, Zhongda Hospital, School of Medicine, Southeast University, 210009 Nanjing, Jiangsu, China; ^4^Department of Obstetrics and Gynecology, Zhongda Hospital, Southeast University, 210009 Nanjing, Jiangsu, China

**Keywords:** postoperative pain, surgery, psychological symptoms, anxiety, depression

## Abstract

**Background:**

Postoperative pain is not only a primary source of physical discomfort but also a significant risk factor that can trigger or exacerbate mental and psychological symptoms such as anxiety and depression. This study aimed to explore the interrelationship between postoperative pain and mental psychological symptoms, such as anxiety and depression, and analyse the risk factors influencing postoperative mental psychological symptoms.

**Methods:**

This study retrospectively collected clinical data of 411 surgical patients from March 2023 to April 2025 through an electronic medical record system. The Numeric Rating Scale (NRS) was used to assess preoperative and postoperative pains during rest and exercise. Postoperative pain was defined as a resting or exercise-evoked NRS score > 3 (moderate to severe pain). The Hospital Anxiety and Depression Scale (HADS) was used to assess anxiety (Hospital Anxiety and Depression Scale-Anxiety [HADS-A]) and depression (Hospital Anxiety and Depression Scale-Depression [HADS-D]). The relationship between postoperative pain and psychological symptoms, such as anxiety and depression, was explored through Spearman's correlation analysis and univariate and multivariate logistic regression analysis.

**Results:**

After surgery, 59.12% (243/411) of patients had chronic postsurgical pain (CPSP), 36.25% (149/411) had anxiety symptoms (HADS-A > 7) and 31.63% (130/411) had depression symptoms (HADS-D > 7). Regarding surgical specialty, orthopaedic surgery patients had the highest proportion of CPSP, accounting for 63.64% (91/143) of orthopaedic cases, which represented 22.14% (91/411) of the total cohort. Gynaecologic surgery patients showed the highest rates of postoperative anxiety (41.76%, 38/91) and depression (45.05%, 41/91) within their specialty. After surgery, the average resting and exercise NRS scores of patients were significantly reduced compared with preoperative levels (both *p* < 0.01). However, the average HADS-A and HADS-D scores were significantly higher than before surgery (*p* < 0.01). Spearman's correlation analysis showed that postoperative resting NRS score was positively correlated with exercise NRS, HADS-A and HADS-D scores (*p* < 0.01). The correlation between HADS-A score and HADS-D score was not strong, whereas the correlation between exercise NRS score and these psychological indicators was strong. Multivariate logistic regression analysis showed that postoperative pain is an independent risk factor for the occurrence of mental symptoms.

**Conclusions:**

A statistically significant mutual association exists between postoperative pain and psychological symptoms in surgery.

## Introduction

The total number of surgical procedures performed worldwide each year has exceeded 300 million, but about 60%–70% of patients still suffer from moderate-to-severe pain after surgery. This common postoperative pain problem considerably reduces the quality of life of patients, delays their physiological function recovery process and increases the burden on the medical system, thus posing a serious challenge to the rational allocation and utilisation of overall medical resources [1, 2]. In recent years, with the continuous deepening of the understanding on pain mechanisms, the academic community has gradually realised that postoperative pain is not a single dimensional physiological response but a multidimensional, dynamic subjective experience that integrates physiological, psychological and sociocultural factors. In this complex network, the psychological state of patients, especially depression and anxiety, is increasingly prominent as an important controllable factor affecting postoperative pain perception, persistence and even deterioration. Its mechanism of action in pain formation and chronicity is receiving widespread attention [3].

The International Association for the Study of Pain (IASP) defines pain as “an unpleasant feeling and emotional experience associated with actual or potential tissue damage” and a protective physiological or pathophysiological response to mechanical, chemical or thermal stimuli, with common triggers such as surgical trauma [4]. A notable detail that if postoperative pain is not well controlled, it may further evolve into chronic postsurgical pain (CPSP). Research has shown that approximately 10%–70% of patients undergoing major surgeries, such as heart, chest, gynaecological and spinal procedures, develop CPSP [5]. The key factors that affect the degree of postoperative pain include patient age; psychological state, such as anxiety; preoperative pain situation; and medication history [6]. With pain being recognised as the fifth major vital sign and the continuous increase in global surgical volume, poor postoperative pain control and its potential psychological complications have become urgent issues that need to be addressed in surgical clinical practice [7].

Mental health disorders are common in surgical patients and closely related to the increase in incidence rate and mortality [8]. Anxiety and depression are important predictive factors for postoperative pain and the main causes of disability worldwide. They are not only core challenges in the field of mental health but also complex issues that profoundly affect individual quality of life, social productivity and public health systems [9]. Clinical studies have revealed a high degree of comorbidity between chronic pain and psychological symptoms. The comorbidity rate of depression and anxiety is particularly prominent amongst patients with chronic pain, with large-scale studies showing that about 20%–40% of adult patients suffer from both diseases simultaneously [10]. Pain is a subjective experience influenced by multiple factors such as psychology, society, culture and emotions. Inadequate pain control may delay early postoperative functional recovery and even increase the risk of chronic pain. The differences in pain and analgesic responses amongst individuals further increase the difficulty of postoperative pain management. Research has shown that depression is associated with acute pain and increased pain perception [11]. Meanwhile, anxiety is regarded as a substantial cause of postoperative pain, it can lower pain thresholds and prolong pain duration [12].

Although existing evidence supports the correlation between pain and psychological symptoms, most studies only focus on the relationship in a single direction or are limited to specific types of surgeries. Therefore, this study aims to provide more comprehensive evidence for clinical prevention and treatment by retrospectively analysing the close relationship between postoperative pain and psychological symptoms in patients undergoing different surgical procedures.

## Materials and Methods

### Subjects

**Fig. 1.**
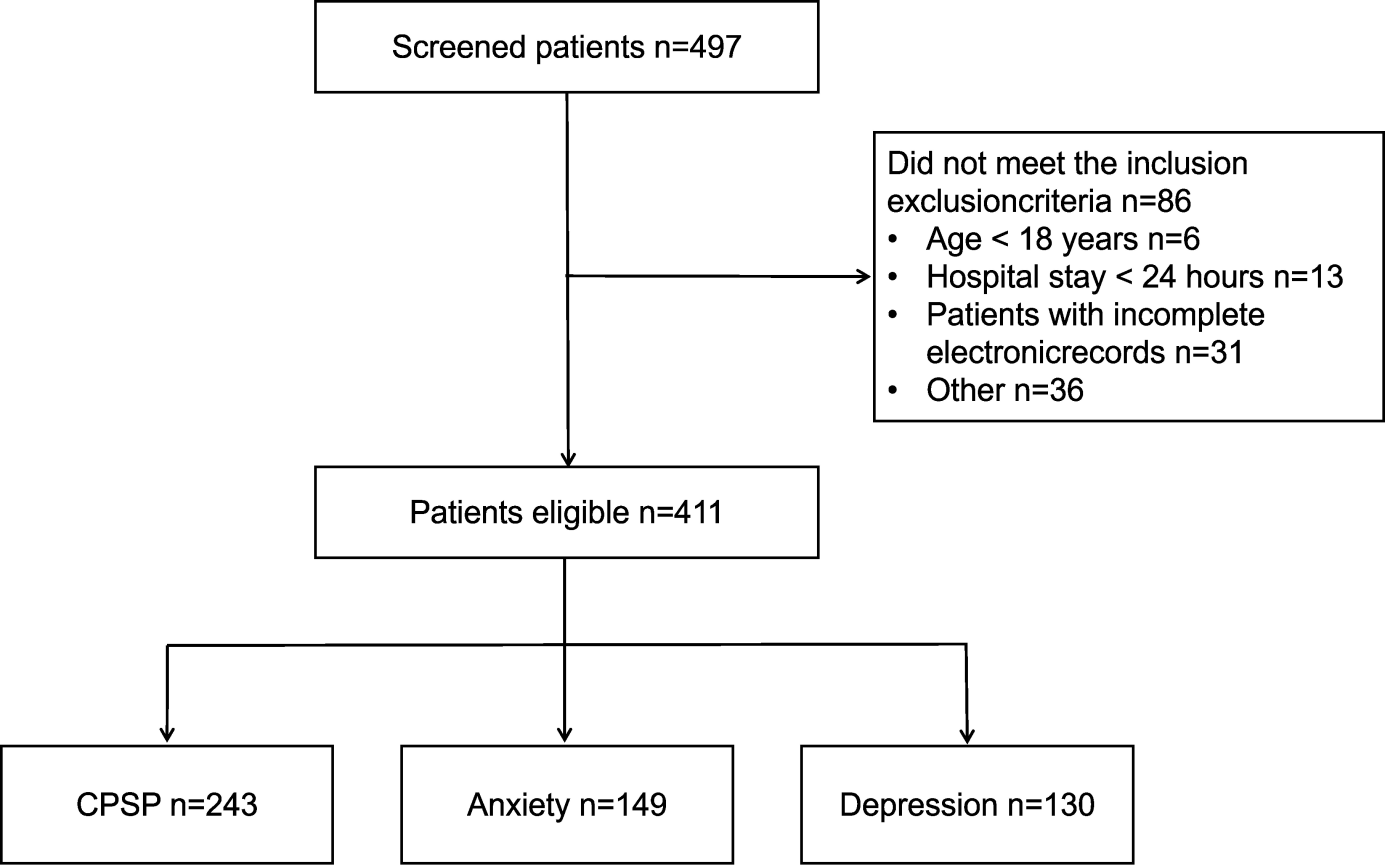
**Participant information**. CPSP, chronic postsurgical pain.

This study adopted a retrospective cohort design. Clinical data of 411 patients who underwent surgical treatment in our hospital from March 2023 to April 2025 were extracted from the electronic medical record system. All postoperative assessments, including pain (Numeric Rating Scale [NRS]), anxiety and depression (Hospital Anxiety and Depression Scale [HADS]) and sleep quality (Pittsburgh Sleep Quality Index [PSQI]), were completed by patients at least 3 months after surgery, and these scores were retrospectively collected from the medical records. A numerical scoring system was used to evaluate the pain intensity of patients in their resting state and during exercise before and after surgery. HADS was used to evaluate the patient’s anxiety state (Hospital Anxiety and Depression Scale-Anxiety [HADS-A]) and depression state (Hospital Anxiety and Depression Scale-Depression [HADS-D]) separately. Spearman’s correlation analysis was conducted to investigate the correlation between postoperative pain severity and psychological symptoms, followed by univariate and multivariate logistic regression analyses to clarify whether postoperative pain, anxiety and depression symptoms are independent influencing factors and to control and correct for possible confounding variables. The participant information is depicted in Fig. 1.

### Inclusion and Exclusion Criteria

Inclusion criteria: (1) admitted to our hospital ward for treatment after undergoing surgical procedures, (2) in the nonpregnancy period, (3) age ≥18 years old and (4) complete and accessible electronic medical records.

Exclusion criteria: (1) neurological disorders that affect pain perception; (2) history of malignant tumours in the past or present; (3) drug abuse or drug dependence; (4) history of chronic pain before surgery; and (5) severe physical illnesses that result in limited mobility, including sequelae of polio, active arthritis, cerebral palsy and other neurological or musculoskeletal disorders that affect daily activities.

### Ethical Statement

This research protocol has been reviewed and approved by the Ethics Committee of Zhongda Hospital, Southeast University (Approval No: 2026ZDSYLL014-P01). The study strictly adheres to the ethical principles outlined in the latest version of the Helsinki Declaration [13]. Written informed consent was obtained from all participants. During the process of data collection and analysis, the personal information of patients was anonymised, and only clinical diagnosis and treatment-related data were retained to ensure the privacy and security of patients.

### Sample Size Calculation

This study followed the formulas in existing literature to estimate the sample size [14]. Under the conditions of a confidence level of 95% and an allowable error of 5%, the minimum sample size required to detect the expected prevalence rate of 70% in the target population was calculated to be 323 cases. This study further expanded the sample size and ultimately included 411 qualified participants to ensure the reliability of the research results and address potential issues such as invalid questionnaires or missing data, thereby providing sufficient statistical support for the accuracy and representativeness of the conclusions drawn in this study.

### Data Collection

Through the hospital electronic medical record system, the basic clinical data of patients included in the study, including age, gender, educational level and type of surgery received, were comprehensively collected. Clinical records related to pain, anxiety and depression were strictly reviewed and extracted from the medical records and systematically organised. This study standardised the selection of cases as follows to minimise the effect of individual differences amongst patients on the research results: patients who underwent common types of surgeries, such as orthopaedic surgery, general surgery and gynaecological surgery, were mainly included, and their duration of illness, anaesthesia management and perioperative management were kept as consistent as possible. All assessments were originally completed by the patients with the use of standardised written instructions as part of routine clinical follow-up. Preoperative evaluations had been conducted within 24 h prior to surgery, and postoperative evaluations had been performed at a minimum of 3 months after surgery. These data were subsequently extracted from the electronic medical record system for the purpose of this retrospective analysis.

The internationally recognised NRS is used to quantitatively evaluate the pain intensity of patients in resting and exercise states at two time points before and after surgery. Postoperative pain was defined as a resting or exercise-evoked NRS score > 3 (moderate to severe pain), consistent with established thresholds for clinically significant postoperative pain. The number of patients meeting this criterion was specifically counted to characterise the pain patient population [15].

HADS is used to assess psychological status. This scale is particularly suitable for patients with physical illnesses. It can effectively identify and quantify the core emotional symptoms of anxiety and depression. This scale consists of two independent dimensions, HADS-A (anxiety dimension) and HADS-D (depression dimension), each of which covers seven items and can quantitatively evaluate the anxiety and depression symptoms of subjects. In this scale, each item is scored 0–3 points, so the total scores of the anxiety subscale (HADS-A) and depression subscale (HADS-D) are both within the range of 0–21 points. Any subscale score > 7 points is defined as clinically significant anxiety or depressive symptoms [16]. For the primary analysis, postoperative mental symptoms were defined as a composite outcome of a HADS-A score > 7 or a HADS-D score > 7.

Sleep assessment: Sleep disturbance was evaluated using PSQI [17], a widely used self-reported questionnaire designed to assess sleep quality and disturbances over the past month. PSQI consists of 19 items grouped into seven components: subjective sleep quality, sleep latency, sleep duration, habitual sleep efficiency, sleep disturbances, use of sleep medication and daytime dysfunction. Each component is scored from 0 to 3, yielding a global score ranging from 0 to 21, with higher scores indicating poorer sleep quality. A global score > 5 is generally considered indicative of clinically significant sleep disturbance [18]. PSQI has demonstrated good reliability and validity in clinical and general populations, including surgical patients (Cronbach’s α > 0.80 in similar studies [19]).

A combination of univariate and multivariate logistic regression analysis strategies was employed to further explore the relationship between postoperative pain and psychological symptoms. Variables with statistical significance in the univariate analysis were selected and then incorporated into a multivariate logistic regression model to control for the influence of potential confounding factors and identify independent associations between postoperative pain and psychological symptoms. Spearman’s correlation analysis was used to evaluate the linear correlation of each score, providing preliminary basis for regression analysis. Covariates were selected a priori based on clinical reasoning to account for potential confounding. These included preoperative pain severity (preoperative NRS scores at rest and during movement), preoperative psychological status (HADS-A and HADS-D scores as continuous variables), history of psychiatric disorders, type of anaesthesia, duration of surgery, postoperative analgesic regimen (opioid vs. non-opioid) and presence of postoperative complications.

### Statistical Analysis

Data were analysed using SPSS 26 software (IBM Corp., Armonk, NY, USA). Measurement data that conformed to a normal distribution are expressed as mean ± standard deviation. Paired t-test was used for within-group comparisons (e.g., preoperative vs. postoperative), and independent *t*-test was used for inter-group comparisons where applicable. For paired data that did not satisfy the normality assumption, the Wilcoxon signed-rank test (a non-parametric method) was used for within-group comparisons. Count data are presented in terms of number of cases and percentage [n (%)], and comparison between groups was conducted using chi square test. The dependent variable for logistic regression was defined as the presence of mental symptoms, operationalised as a HADS subscale score > 7 on either the anxiety (HADS-A) or depression (HADS-D) dimension, indicating clinically significant anxiety or depressive symptoms. Missing data for covariates were handled using multiple imputation by chained equations under the missing-at-random assumption. Twenty imputed datasets were generated, and the results were pooled using Rubin’s rules. By comprehensively using single factor and multiple factor logistic regression analyses, supplemented by Spearman’s correlation analysis, this study systematically explored the independent association and interaction between postoperative pain and psychological symptoms. All statistical tests were two-sided, and the difference was considered statistically significant with *p* < 0.05.

## Results

### Baseline Characteristics

Amongst the 411 patients included, the average age was (48.48 ± 13.95) years, and the male-to-female ratio was comparable, with males accounting for 51.09% and females accounting for 48.91%. The cases mainly come from surgical patients in orthopaedics, general surgery and gynaecology. In addition, the educational level of patients was generally low, concentrated in high school and below stages. Preoperative psychological assessment showed that 14.84% and 12.90% of patients had symptoms of anxiety and depression, respectively (Table 1).

**Table 1.** **Baseline characteristics**.

Variables		n (%) / Mean ± SD
Gender, n (%)		
	Male	210 (51.09%)
	Female	201 (48.91%)
Age (years, mean ± SD)		48.48 ± 13.95
Educational level, n (%)		
	University	109 (26.52%)
	High school	128 (31.14%)
	Junior high school and below	151 (36.74%)
	Illiterate	23 (5.60%)
Type of surgery, n (%)		
	Orthopaedic surgery	143 (34.79%)
	General surgery	122 (29.68%)
	Gynaecologic surgery	91 (22.14%)
	Other	55 (13.38%)
Preoperative pain and psychological status n (%)		
	Resting NRS > 3	249 (60.58%)
	Movement-evoked pain NRS > 3	232 (56.45%)
	HADS-A > 7	61 (14.84%)
	HADS-D > 7	53 (12.90%)
	Preoperative sleep disturbance (PSQI > 5)	173 (42.09%)
	Use of pain medication in past 12 h	317 (77.13%)
	Preoperative course of Illness duration > 1 month	59 (14.36%)

Note: NRS, Numeric Rating Scale; HADS-A, Hospital Anxiety and Depression Scale-Anxiety; HADS-D, Hospital Anxiety and Depression Scale-Depression; PSQI, Pittsburgh Sleep Quality Index; SD, standard deviation.

### Occurrence of Postoperative Pain and Mental Symptoms

In this study, postoperative pain was assessed at a minimum of 3 months after surgery, consistent with the widely accepted definition of CPSP proposed by IASP. Patients with pre-existing chronic pain prior to surgery were excluded on the basis of the exclusion criteria. Amongst the 411 patients included, the incidence of CPSP was 59.12% (243 cases). Overall, the incidence rates of postoperative anxiety and depression were 36.25% and 31.63%, respectively. In terms of surgical type, gynaecological surgery patients had the highest incidence of anxiety (41.76%) and depression (45.05%). In addition, the incidence of postoperative sleep disturbance (PSQI > 5) was 51.38%, indicating that sleep problems are common in this cohort. These findings suggest that long-term psychological and physiological health issues after surgery are very common and that they require attention comparable to that given to the surgical procedure itself (Table 2).

**Table 2.** **Occurrence of postoperative pain and mental symptoms**.

Type of surgery	Number	CPSP	Anxiety	Depression	Sleep disturbance
Orthopaedic surgery	143	91 (63.64%)	54 (37.76%)	39 (27.27%)	85 (59.44%)
General surgery	122	66 (54.10%)	42 (34.43%)	37 (30.33%)	62 (50.82%)
Gynaecologic surgery	91	57 (62.64%)	38 (41.76%)	41 (45.05%)	50 (54.95%)
Other	55	29 (52.73%)	15 (27.27%)	13 (23.64%)	27 (49.09%)
Total	411	243 (59.12%)	149 (36.25%)	130 (31.63%)	211 (51.38%)

Note: CPSP, chronic postsurgical pain; PSQI, Pittsburgh Sleep Quality Index.

### Comparison of Preoperative and Postoperative Pains and Psychological Assessment Results

After undergoing surgery, the patients’ pain levels in resting and exercise states were significantly reduced compared to preoperative levels (*p* < 0.01), indicating that surgical plans have static and dynamic efficacy in relieving pain. In addition, the HADS-A and HADS-D scores showed a significant upward trend compared with their preoperative baseline levels (*p* < 0.01). This result clearly suggests that surgical intervention may be associated with increased anxiety and depression in patients (Table 3).

**Table 3.** **Comparison of preoperative and postoperative pains and psychological assessment results**.

Index	Preoperative (n = 411)	Postoperative (n = 411)	Z	*p*
Resting NRS	4.00 (2.00, 6.00)	4.00 (1.00, 5.00)	4.011	<0.001
Movement-evoked pain NRS	5.00 (2.00, 7.00)	4.00 (1.00, 7.00)	3.244	0.001
HADS-A	4.00 (2.00, 6.00)	5.00 (3.00, 10.00)	–4.395	<0.001
HADS-D	4.00 (2.00, 6.00)	5.00 (2.50, 9.00)	–3.779	<0.001

Note: For non-normally distributed data presented in this table, the Wilcoxon signed‑rank test was used. NRS, Numeric Rating Scale; HADS-A, Hospital Anxiety and Depression Scale-Anxiety; HADS-D, Hospital Anxiety and Depression Scale-Depression.

### Correlation Analysis of Postoperative Pain and Psychological Indicators

The resting NRS score and movement-evoked pain NRS score can indicate the degree of pain in surgical patients. Spearman’s correlation analysis showed that resting NRS score was highly positively correlated with movement-evoked pain NRS score (*ρ* = 0.899, *p* < 0.001). Resting NRS score was significantly positively correlated with HADS-A score (*ρ* = 0.197, *p* < 0.001) and HADS-D score (*ρ* = 0.210, *p* < 0.001), indicating a certain relationship between pain level and anxiety and depression, but the correlation coefficient was low, suggesting limited actual correlation strength. In addition, the correlation between exercise NRS score and these psychological indicators was strong (HADS-A: *ρ* = 0.262, *p* < 0.001; HADS-D: *ρ* = 0.326, *p* < 0.001). A moderate positive correlation was found between HADS-A and HADS-D scores (*ρ* = 0.568, *p* < 0.001), indicating a clear co-occurrence trend of anxiety and depression symptoms in the study population. Individuals with more severe anxiety symptoms often had more prominent depression symptoms (Table 4).

**Table 4.** **Correlation analysis of postoperative pain and psychological indicators**.

Index	Resting NRS	*p*	Movement-evoked pain NRS	*p*	HADS-A	*p*	HADS-D
Resting NRS	1.000						
Movement-evoked pain NRS	0.899	<0.001	1.000				
HADS-A	0.197	<0.001	0.262	<0.001	1.000		
HADS-D	0.210	<0.001	0.326	<0.001	0.568	<0.001	1.000

Note: NRS, Numeric Rating Scale; HADS-A, Hospital Anxiety and Depression Scale-Anxiety; HADS-D, Hospital Anxiety and Depression Scale-Depression.

### Analysis of Risk Factors for Postoperative Mental Symptoms

Multivariate logistic regression analysis showed that after this study adjusted for potential confounding factors, such as age and gender, postoperative pain (OR (95% CI) = 2.749 (1.726–4.378), *p* < 0.001) remained an independent risk factor for mental symptoms. This finding indicates that these factors, after excluding other interferences, still have a considerable effect on the occurrence of mental health disorders (Table 5).

**Table 5.** **Analysis of risk factors for postoperative mental symptoms**.

Variables		Univariable analysis					Multivariable analysis				
		β	SE	Z	*p*	OR (95% CI)	β	SE	Z	*p*	OR (95% CI)
Postoperative pain											
	No					1.000 (Reference)					1.000 (Reference)
	Yes	0.781	0.215	3.631	<0.001	2.184 (1.433–3.329)	1.011	0.237	4.259	<0.001	2.749 (1.726–4.378)
Age											
	≤ 60 years					1.000 (Reference)					1.000 (Reference)
	> 60 years	0.175	0.243	0.719	0.472	1.191 (0.739–1.919)	0.468	0.266	1.762	0.078	1.597 (0.949–2.688)
Gender											
	Male					1.000 (Reference)					1.000 (Reference)
	Female	0.204	0.212	0.962	0.336	1.226 (0.809–1.857)	0.382	0.226	1.689	0.091	1.466 (0.941–2.284)
Preoperative HADS-A > 7											
	No					1.000 (Reference)					1.000 (Reference)
	Yes	0.799	0.368	2.169	0.030	2.223 (1.080–4.576)	0.449	0.419	1.071	0.284	1.566 (0.689–3.561)
Preoperative HADS-D > 7											
	No					1.000 (Reference)					1.000 (Reference)
	Yes	0.876	0.351	2.493	0.013	2.402 (1.206–4.783)	0.665	0.398	1.669	0.095	1.944 (0.891–4.241)
Educational level											
	University					1.000 (Reference)					
	High school	0.049	0.262	0.186	0.852	1.050 (0.628–1.756)					
	Junior high school and below	0.050	0.253	0.200	0.842	1.057 (0.641–1.727)					
	Illiterate	0.426	0.478	0.891	0.373	1.531 (0.600–3.910)					
Preoperative sleep disturbance											
	No					1.000 (Reference)					
	Yes	–0.293	0.213	–1.376	0.169	0.746 (0.491–1.133)					
Postoperative sleep disturbance											
	No					1.000 (Reference)					
	Yes	–0.055	0.211	–0.261	0.794	0.946 (0.625–1.432)					
Preoperative course of Illness duration > 1 month											
	No					1.000 (Reference)					
	Yes	–0.354	0.291	–1.216	0.224	0.702 (0.397–1.241)					
Use of pain medication in past 12 h											
	No					1.000 (Reference)					
	Yes	–0.341	0.262	–1.302	0.193	0.711 (0.426–1.188)					

Note: OR, odds ratio; CI, confidence interval; SE, standard error; HADS-A, Hospital Anxiety and Depression Scale-Anxiety; HADS-D, Hospital Anxiety and Depression Scale-Depression.

## Discussion

Surgical procedures, as an important means of treating diseases, are increasingly receiving widespread attention for postoperative management. Postoperative acute pain is one of the most common complications of surgical procedures [20]. If not well controlled, it directly affects patients’ early mobilisation and functional recovery, prolongs hospitalisation time and may progress to chronic postoperative pain, thereby seriously damaging patients’ quality of life [21]. In recent years, with the deepening of the “biopsychosocial” medical model, the mental health status of perioperative patients has become a research focus. Numerous clinical observations have found a close correlation between severe postoperative pain and the onset of psychological symptoms such as anxiety and depression [22, 23, 24]. Therefore, in-depth exploration of the statistical association between postoperative pain and psychological symptoms in surgery is of great clinical importance for developing comprehensive perioperative pain management and psychological intervention strategies and improving patients’ long-term prognosis.

Studies have shown that approximately 70% of individuals in the patient population requiring surgical treatment experience varying degrees of pain, and 38% of patients exhibit considerable anxiety and stress. The incidence of pain is more common in the postoperative stage than before surgery, whereas anxiety is more concentrated in the preoperative stage [15]. The incidence rate of post-operative anxiety and depression in the present study was higher than that before surgery, may be because this study focused on the evaluation of mental state in the postoperative rehabilitation stage, and patients may face multiple stressors, such as pain management, uncertainty of functional recovery and medical costs, at this stage. Unlike preoperative fear of the unknown, postoperative anxiety often stems from the gap amongst the actual rehabilitation process and expectations, the effect of medication side effects on emotions and the psychological adaptation problems caused by a temporary lack of social roles [25]. A study has shown that the anxiety scores of patients after gynaecological surgery significantly decrease, but depression scores may increase [26]. However, the findings of the present study demonstrated a different trend. The patients’ anxiety and depression scores increased postoperatively. The reason for this difference may be related to the fact that the types of surgeries included in this study are more diverse and the patient population is more heterogeneous, thus reflecting the complexity of the effect of different surgical types on patients’ emotional states.

So far, substantial heterogeneity and inconsistent conclusions continue to exist in the literature reports on the factors affecting postoperative pain in surgery. Psychological factors, especially anxiety and depression, are considered as potential important predictive variables. However, their role in postoperative pain remains unclear due to insufficient preoperative systematic evaluation of anxiety and depression as well as conflicting results between different studies [27]. Multiple studies have shown that depression and anxiety significantly increase the risk of postoperative pain and complications in patients [28, 29, 30]. The present study showed that increased postoperative pain levels were associated with an increased likelihood of postoperative anxiety and depression, supporting the interrelationship between these factors. However, the directionality or causality of these associations could not be determined due to the retrospective nature of this study. Although some existing studies have limited sample sizes and differences in covariate control, the overall evidence still supports the conclusions of the present study. Meanwhile, the pain, depression and anxiety data involved in this study were analysed on the basis of patients’ self-reported postoperative scale scores [31], and this methodological feature should be taken into account when interpreting the results. Postoperative moderate-to-severe pain has been proven to be an important independent predictor of anxiety and depression symptoms in patients after surgery. The present study further confirmed a significant correlation between postoperative pain intensity and emotional disorders such as anxiety and depression. The higher the degree of pain, the higher the risk of psychological distress, such as anxiety and depression, in patients. This finding is consistent with the conclusions of existing literature [32]. From a clinical perspective, the observed small-to-moderate correlations between pain intensity and psychological symptoms warrant attention. In multifactorial conditions such as postoperative recovery, even modest statistical associations can translate into meaningful differences in patient-reported outcomes, particularly when considering the cumulative effect across populations.

Preoperative anxiety is a common psychological state amongst patients, and existing research generally suggests a correlation between it and postoperative pain, which may affect postoperative pain intensity and analgesic needs through psychological mechanisms. Multiple studies have shown that preoperative high anxiety is not only a trigger for increased postoperative pain but also an important predictor of the development of moderate-to-severe pain [33, 34, 35]. Postoperative pain is significantly correlated with anxiety and depression symptoms. Although different studies use different scales, the conclusions are consistent [36]. However, some studies did not show a significant association between anxiety symptoms and pain levels, may be due to insufficient statistical power, sample heterogeneity or the presence of uncontrolled confounding factors such as differences between populations of patients with acute and chronic symptoms. In addition, anxiety and pain may mutually influence each other, and postoperative pain itself is influenced by factors such as age and type of surgery [4]. Therefore, when exploring the relationship between the two, comprehensively considering multiple variables and differences in the research design is necessary.

Surgical intervention, as an important clinical intervention, may induce psychological symptoms, such as anxiety and depression, in patients whilst treating diseases [37]. Multiple studies have further indicated that long-term or chronic use of opioid drugs significantly increases the risk of developing new depression and/or clinically significant anxiety [38, 39]. Opioids still have a place in the management of specific types of chronic pain, and the association between their use and depression and anxiety suggests that pain treatment strategies need to consider the psychological effect. Although the pain assessment tools differed from those in previous studies, the conclusions drawn in the present study are consistent, i.e., pain itself is an important risk factor for depression and clinically significant anxiety, suggesting that high attention should be paid to the psychological health status of patients in pain management.

In addition, studies have shown that the close relationship between emotional disorders and acute pain is increasingly being recognised by the academic community as being risk factors and influencing each other. Specifically, depression and anxiety often accompany an increased perception of pain severity, and the persistent presence of acute pain may further impair an individual’s ability to regulate emotions [40]. However, research on the interaction between emotional disorders and pain during the rehabilitation process after surgery is still relatively scarce. The present study not only confirmed that postoperative anxiety and depression are independently associated with increased postoperative pain but also revealed that preoperative psychological state and initial pain level are important factors associated with postoperative psychological symptoms.

### Meaning and Innovation

This retrospective study confirmed a statistically significant mutual association between postoperative pain and psychological symptoms such as anxiety and depression. Compared with traditional models that are limited to identifying one-way risk factors, this study adopted a multivariate logistic regression method to preliminarily construct a two-way risk prediction model, which has certain innovation. In addition, the study preliminarily revealed differences in the incidence of CPSP and psychological symptoms amongst different surgical specialties (such as orthopaedics and gynaecology), suggesting that future intervention strategies should be tailored to the characteristics of patients in different departments, and more targeted “individualised” and “specialised” management plans should be developed, providing a basis for further research on specialty oriented perioperative psychosomatic comprehensive management.

### Research Limitations

However, this study has several limitations. Firstly, the study adopted a retrospective analysis design, which is constrained by the completeness and accuracy of historical data during data collection and analysis. This design type cannot actively control variables and follow-up time series like prospective studies, hence its inherent limitations in inferring causal relationships. The association conclusions need to be further validated through more rigorous designs in the future. Secondly, the sample size included in this study is relatively limited. Although efforts have been made to expand the sample size under existing conditions, it may still affect the statistical power, resulting in insufficient detection ability of some weak correlations, and reduce the stability and extrapolation of multivariate analysis results. Thirdly, although the Methods section stated that preoperative HADS-A and HADS-D scores were considered as covariates, these variables were not included in the final multivariable logistic regression model presented in Table 5 due to modelling constraints. Consequently, the current multivariable analysis did not adjust for preoperative anxiety or depression status, which are amongst the strongest known confounders for postoperative psychological symptoms. The failure to adjust for these variables is an important limitation of the present study. In addition, in terms of evaluation tools, HADS was chosen as a screening tool for emotional symptoms. The initial design goal of this scale was to quickly identify patients’ anxiety and depression symptoms in a comprehensive hospital environment. Its entries were deliberately set to avoid overlapping with common physical symptoms, such as fatigue and insomnia, to enhance the specificity of emotional problem screening in comorbid populations. A notable detail that the depression subscale of HADS mainly focuses on two aspects: core emotional states of depression and lack of pleasure. Moreover, its structure does not strictly correspond to all diagnostic criteria for severe depression in the DSM-5-TR revised version. However, numerous studies have shown that the sensitivity and specificity of HADS in screening for severe depression are comparable to those of the widely used Patient Health Questionnaire-9 (PHQ-9), and they have certain clinical applicability and reference value [41]. Future research needs to combine diagnostic interviews and multidimensional assessment tools, which could help to more comprehensively and accurately capture the emotional pathological characteristics of research subjects.

## Conclusions

A statistically significant mutual association was found between postoperative pain in surgery and psychological symptoms such as anxiety and depression. However, the correlation between pain intensity and anxiety or depression scores was weak to moderate.

## Availability of Data and Materials

The data supporting the findings of this study can be obtained from the corresponding author, upon request.
